# Biological effect of acupuncture on peripheral facial paralysis

**DOI:** 10.3389/fneur.2025.1516904

**Published:** 2025-04-24

**Authors:** Wenwen Duan, Dan Chen, Zubo Huang, Yue Zeng, Shanshan Liu, Chao Wang, Hao Zhou

**Affiliations:** ^1^School of Acupuncture-Moxibustion and Tuina, Chengdu University of Traditional Chinese Medicine, Chengdu, China; ^2^Sichuan Clinical Research Center for Sub-Health, Sichuan Integrative Medicine Hospital, Chengdu, China

**Keywords:** acupuncture, peripheral facial paralysis, neuroinflammation, molecular neurobiology, biological effect

## Abstract

Peripheral facial paralysis is the dominant treatment disease of acupuncture. A large number of studies have proved the effectiveness of acupuncture in the treatment of peripheral facial paralysis. However, the underlying biological effect remains in an exploratory phase. This article will sort out and summarize the existing research mechanisms from the following aspects: inflammatory response, immune regulation, neurotransmitters, immune response, facial microcirculation, oxidative stress, changes in nerve structure and function, specificity of acupoints, acupuncture intervention time, and other potential mechanisms aiming to provide a scientific foundation for the role of acupuncture in the treatment of peripheral facial paralysis. Furthermore, the review discusses future directions for mechanistic research based on existing findings.

## 1 Introduction

Peripheral facial paralysis is a prevalent clinical disorder characterized by inflammation and edema in the subnuclear motor neurons, resulting in facial nerve paralysis (1). In modern medicine, it is classified as a neurological disease (2). On average, 15–23 per 100,000 individuals are affected annually by peripheral facial paralysis, with a 12% likelihood of recurrence (3). Clinical symptoms of peripheral facial paralysis typically include loss of forehead wrinkles on the affected side, inability to raise the eyebrow, incomplete eyelid closure, the frequent tears on the eyelids, inability to puff the cheeks, and deviation of the mouth corner toward the healthy side (4). Patients with peripheral facial paralysis may also have difficulties in drinking water, eating, and other activities (5). Due to the paralysis of one side of the facial muscles, patients often have psychological problems such as anxiety and depression, resulting in social disorders and decreased quality of life (6).

According to the clinical practice guidelines of evidence-based acupuncture, peripheral facial paralysis is one of the dominant diseases of acupuncture (7). In 2023, the' Guidelines for the Treatment of Idiopathic Facial Paralysis' issued by the Japanese Facial Nerve Research Society recommended acupuncture as an effective intervention for the treatment of peripheral facial paralysis (8). This recommendation is based on the potential benefits of acupuncture in improving facial nerve function and promoting facial paralysis recovery. A bibliometric study (9) suggests that acupuncture can promote the functional recovery of facial nerves to varying degrees by improving some inflammatory factors. The early intervention of acupuncture does improve the prognosis of facial paralysis to a great extent (10). This is because acupuncture intervenes in the acute phase can accelerate the promotion of axon growth and improve neurotrophic nutrition (11, 12). Although acupuncture has shown certain efficacy in the treatment of peripheral facial paralysis, the mechanism behind it is still not fully understood. In addition, there are relatively few review studies on the mechanisms of acupuncture for peripheral facial paralysis. Therefore, this paper aims to explore the mechanism of acupuncture in the treatment of peripheral facial paralysis by combing the existing relevant literature (Table 1).

**Table 1:** Comprehensive overview of clinical and animal model studies for acupuncture's biological effects.

**Author**	**Publication year**	**Research object**	**Intervention measures**	**Observation indicators**	**Reference**
Chengcheng Han, et al.	2024	Patients with peripheral facial paralysis	Acupuncture	hs-CRP IL-6 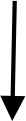	(14)
Luyao Zhang, et al.	2021	Acute pancreatitis mice	Electroacupuncture	α7nAChR TNF-α IL-1β IL-6 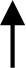 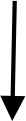	(17)
Jiang Zhao, et al.	2020	Patients with peripheral facial neuritis	Electroacupuncture	TLR4 NF-κBp65 protein 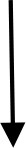	(20)
Xue Xiao, et al.	2023	Rats with primary dysmenorrhea	Electroacupuncture	PGE_2_ PGF_2α_ TLR4 NF-κBp65 IL-1β IL-18 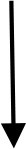 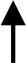	(21)
Shuangning Song, et al.	2018	Mice with acute colitis	Electroacupuncture	IL-1β TNFα IL-6 IL-12 IL17 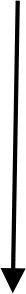	(24)
Yanyu Xu, et al.	2017	Children with facial neuritis	Mouse nerve growth factor combined with acupuncture	IL-17 IL-6 TGF-β1 IL-10 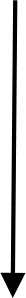 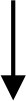	(25)
Jun-peng Yao, et al.	2024	Facial nerve injury in rats	Electroacupuncture	GDNF PI3K mTOR Beclin-1 LC3 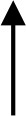 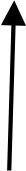	(36)
Jing Fei, et al.	2018	Facial nerve crush injury	Electroacupuncture	GDNF N-cadherin 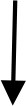	(37)
Na Zang, et al.	2023	Wind-cold type idiopathic facial nerve paralysis in acute stage patients	Electroacupuncture	GDNF NGF 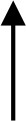	(38)
Cheng Tang, et al.	2024	Patients with acute-phase idiopathic facial nerve palsy	Alternating warm needle acupuncture combined with nerve growth factor from rats	GDNF NGF SOD 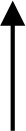	(39)
Sun Yunhua, et al.	2011	Acute facial nerve injury rabbit model	Electroacupuncture	CNTFR 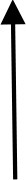	(42)
Sun Zhongren, et al.	2006	Rabbits with peripheral facial nerve injury	Electroacupuncture	BNDF mRNA 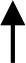	(44)
Zhou Shuxin, et al.	2018	Patients with facial paralysis	Acupuncture	Ig A Ig G Ig M 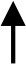	(48)
Weiping Liang	2023	Patients with peripheral facial paralysis	Acupuncture	IgG IgM 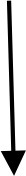	(49)
Sun Hui	2021	Patients after colorectal cancer radical operation	Warm-needle moxibustion	CD3+ CD4+ TNF-α IL-6 CRP 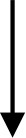 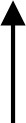	(50)
Qianqian Cui, et al.	2024	Patients in the acute phase of peripheral facial paralysis	Acupuncture plus moxibustion	hs-CRP WBC NLR PLR 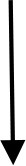	(53)
Jia Shi, et al.	2024	Acute lung injury model in mice	Electroacupuncture	HO-1 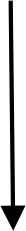	(61)
Jianguo Li, et al.	2024	CUMS Rats	Acupuncture	CAT SOD GSH-Px 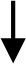	(63)
Lv Shanguang, et al.	2016	Rat model of ischaemic facial paralysis	Acupuncture	NO ET 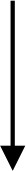 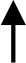	(76)
Jin Liu	2016	Patients with acute idiopathic facial nerve palsy	“Qianzheng San” with warm acupuncture	EPCs 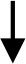	(77)

### 1.1 Acupuncture improves facial inflammatory response

**Figure 1:**
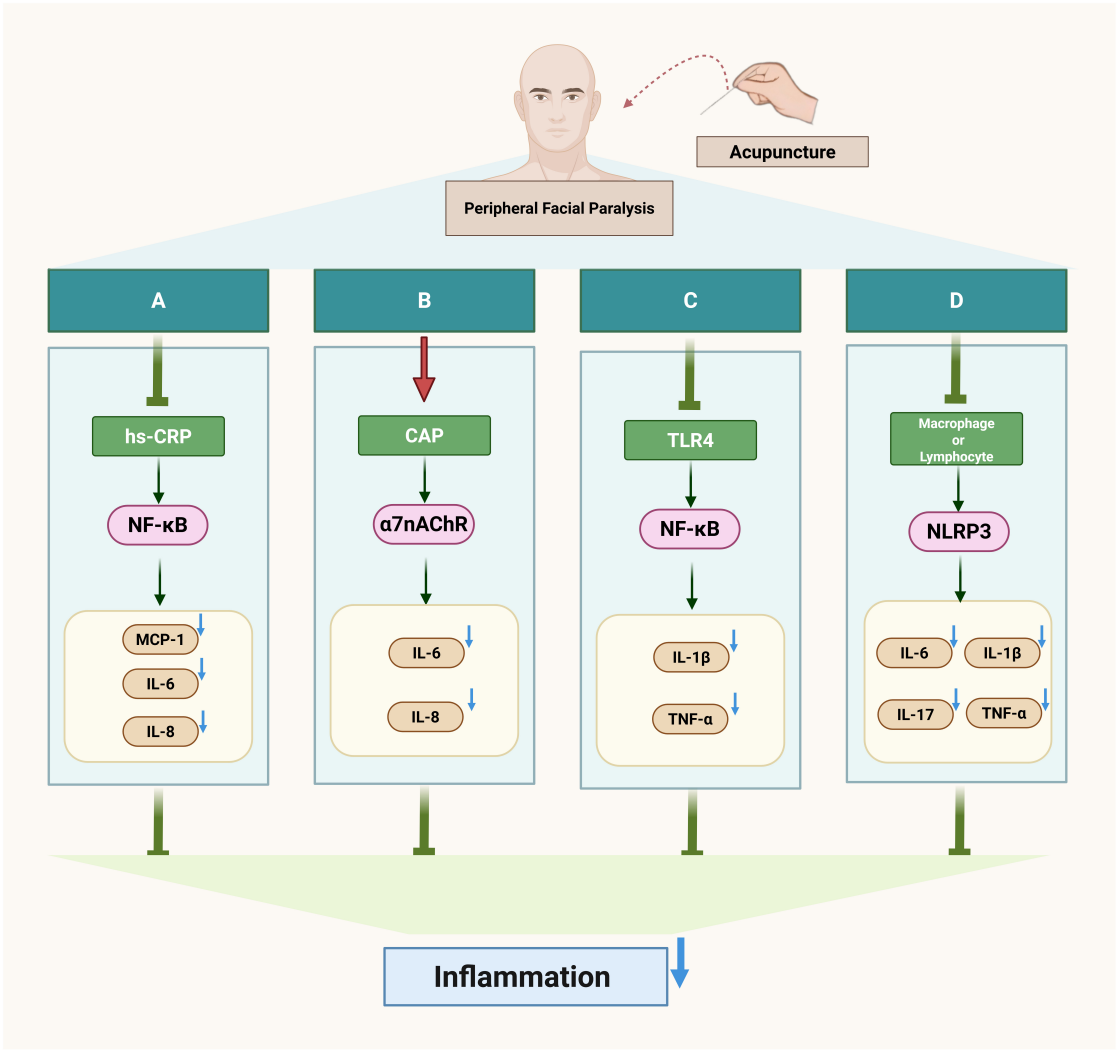
The anti-inflammatory process of acupuncture treatment of peripheral facial paralysis.

Inflammation is currently recognized as the pathogenesis of peripheral facial paralysis. After exiting the medulla oblongata from the cerebral bridge, the facial nerve traverses the narrow passage between the internal auditory canal and the mastoid foramen of the stem, where inflammatory pathogenic factors infiltrate the facial nerve, leading to demyelination of myelinated axons on the nerve fibers and the development of facial neuritis (13). Acupuncture can improve facial inflammation of peripheral facial paralysis by regulating inflammatory factors (Figure 1).

#### 1.1.1 Acupuncture regulates the balance of anti-inflammatory and pro-inflammatory factors

Hs-CRP is a biomarker of inflammation, while mCRP is its subtype with pro-inflammatory properties. mCRP activates endothelial cells, releases pro-inflammatory cytokines such as MCP-1, IL-6, and IL-8, and promotes downstream inflammatory responses through the NF-κB pathway, thereby participating in the onset of inflammation (14, 15). The ratio of C-reactive protein to albumin in the serum of patients with facial neuritis is elevated, indicating the presence of an inflammatory response (16). Acupuncture can reduce the content of inflammatory factor hs-CRP in patients with peripheral facial paralysis and improve the inflammatory state (17). Acupuncture can also reduce the level of inflammation by regulating macrophages. Macrophages, a type of inflammatory cytokine, exist in two phenotypes: M1, which promotes the secretion of inflammatory cytokines, and M2, which inhibits the inflammatory response (18). Electroacupuncture modulates macrophage polarization by suppressing pro-inflammatory pathways (NLRP3/IL-1β) and activating anti-inflammatory mechanisms (Nrf2/HO-1/IL-10), effectively shifting macrophages from the M1 to M2 phenotype and improving inflammation (19). In addition, in a study of peripheral facial neuritis, it was found that the expression of Treg cells and Th17 cytokines in the peripheral serum of patients was unbalanced, the anti-inflammatory cytokine IL-10 released by Th17 cells was low, and the pro-inflammatory cytokines IL-6 and IL-17 released by Treg cells were highly expressed (20). However, acupuncture can down-regulate the expression of inflammatory cytokines IL-1β, TNF-α, and IL-6 in serum, inhibit the secretion of TNF-α, IL-17 protein, and mRNA, and promote the levels of serum anti-inflammatory cytokines IL-12 and IL-10 mRNA (21). This suggests that acupuncture exerts its therapeutic effect on peripheral facial paralysis by modulating the balance between pro-inflammatory and anti-inflammatory factors, thereby ameliorating the inflammatory response associated with peripheral facial paralysis.

#### 1.1.2 Acupuncture activates the cholinergic anti-inflammatory pathway (CAP)

The cholinergic system involves neurotransmitters/molecules, acetylcholine (ACh), cholinergic receptors (AChRs), choline acetyltransferase (ChAT), and acetylcholinesterase (AChE) (22). These molecules participate in the body's immune response by binding to receptors on different immune cells, such as T cells, B cells, and lymphocytes, and in the complex cholinergic system, the activation of vagal efferents can mediate the release of acetylcholine from α7 nicotinic acetylcholine receptor (α7nAChR), which makes the expression of some pro-inflammatory cytokines decrease in the body, such as TNF-α, IL-6 and IL-18, forming cholinergic anti-inflammatory pathway (CAP) and alleviating inflammatory response (22, 23). Studies have found that electroacupuncture regulates inflammation by stimulating acetylcholine in the enteric nervous system, acting on α7nAChR expressed on inflammatory cells macrophages, and monocytes, which in turn inhibits the production of pro-inflammatory cytokines (24). Continuous acupuncture for 7 days increased the expression of α7nAChR in hippocampal neurons and decreased the expression of the downstream pro-inflammatory cytokines TNF-α and IL-1β (25). This suggests that acupuncture can regulate multiple downstream pro-inflammatory factors by activating the cholinergic anti-inflammatory pathway (CAP), thereby suppressing inflammation.

#### 1.1.3 Acupuncture inhibits toll-like receptor 4 (TLR4)/NF-kB pathway transmission

A pathological manifestation of facial neuritis is the disappearance of axons on its nerve fibers, and the NF-kB pathway is involved in the initiation, growth, and branching of nerve fiber axons, and promotes myelin formation, and this process effectively aids in myelin sheath regeneration in peripheral facial paralysis, helping restore facial nerve function (26). However, NF-kB is regulated by TLR4, one of the players in inflammation, and TLR4 can activate tumor necrosis factor-receptor-associated factor 6 (TRAF6) via myeloid differentiation factor 88 (MyD88), which activates the downstream NF-κB pathway, followed by phosphorylation of residue S536 on the NF-κBp65 subunit, which allows NF-κB to bind to sites on promoters or enhancers of target genes, exerting inflammatory regulatory effects and leading to inflammation in the facial nerve (27–29). Additionally, patients with peripheral facial paralysis show significantly higher serum levels of tumor necrosis factor-α (TNF-α) and interleukin-1β (IL-1β) compared to healthy individuals, possibly due to the inflammatory cascade triggered by the activation of the TLR4/NF-κB pathway (30). A study has confirmed (31) that electroacupuncture can down-regulate the expression of TLR4 mRNA and protein as well as the level of NF-κBp65 phosphorylation, and effectively inhibit the TLR4/NF-kB pathway conduction in peripheral facial paralysis.

### 1.2 Acupuncture promotes the secretion of neurotrophic factors to repair damaged nerves

The neurotrophic factor family includes nerve growth factor (NGF), brain-derived neurotrophic factor (BDNF), ciliary neurotrophic factor (CNTF), neurotrophic factor (NT-3) (32), and glial cell-derived neurotrophic factor (GDNF) (33). Neurotrophic factors have the effect of repairing peripheral nerve injury. The immediate manifestation of facial nerve injury is paralysis of the expression muscles controlled by it (34). Muscle atrophy occurs due to a decrease in total muscle protein metabolism and myoglycogen content, among other things, when skeletal muscle is removed from the innervation of the nerve (35, 36). The neurotrophic factors secreted by neurons can precisely be used as therapeutic targets for muscle atrophy (37).

#### 1.2.1 Up-regulate nerve growth factor and glial cell line-derived neurotrophic factor (GDNF)

NGF can promote the development, differentiation, regeneration, and repair of central and peripheral neurons, and accelerate myelin repair as well as myelin production, and peripheral facial paralysis is a disease of demyelination of nerve fiber axons, so serum NGF may be a therapeutic target for facial neuritis (38, 39). Acupuncture can protect neurons by up-regulating nerve growth factors and promote axonal regeneration to improve facial paralysis symptoms (40). In addition, studies have found that activation of inositol phospholipid-3-kinase (PI3K) can phosphorylate protein kinase B (AKT), which can effectively protect nerve cells (41). By up-regulating the expression of cell-derived neurotrophic factors, electroacupuncture activates the PI3K-AKT-mTOR signaling pathway, down-regulates the levels of autophagy protein markers Beclin-1 and light chain 3 (LC3), inhibits the body's autophagy level, and promotes the repair of facial neurons (42). The study of Fei et al. confirmed that electroacupuncture can up-regulate the expression of GDNF, improve the degree of nerve injury, and promote neuronal regeneration (43). Similarly, Zang et al. (44) found that acupuncture combined with warm moxibustion in patients with facial nerve paralysis due to wind-cold syndrome increased the expression of GDNF and nerve growth factor (NGF) in serum. Tang et al.'s clinical study also confirmed that warm acupuncture combined with murine nerve growth factor significantly boosted GDNF and NGF levels, further supporting the therapeutic potential of acupuncture in nerve regeneration (45). These findings suggest that acupuncture may play a vital role in neural repair and regeneration through the regulation of neurotrophic factors.

#### 1.2.2 Up-regulate ciliary neurotrophic factor (CNTF)

Ciliary neurotrophic factor (CNTF) is found in glial cells of the peripheral nervous system, where it binds to subunit α of the CNTFR complex, induces heterodimerization of the β-receptor subunit to undergo tyrosine phosphorylation, and activates STAT proteins to migrate to the nucleus and bind to specific DNA, acting as a neuroprotective agent (46). CNTF can also effectively improve facial nerve injury, promote facial nerve axon regeneration, and regulate synaptic plasticity (47). Studies have shown (48) that electroacupuncture can promote the expression of CNTF receptors in rats with acute-phase facial nerve injury, and the up-regulation of its receptor expression level implies an increase in the number of sites that bind to CNTF, which contributes to the protective and regenerative effects of CNTF on the facial nerve.

#### 1.2.3 Up-regulate brain-derived neurotrophic factor (BDNF)

BDNF can protect neuronal survival and participate in the regulation of neuroinflammation. The combination of BDNF and tyrosine kinase (TrkB) receptors can increase the signal transduction of Ca_2_^**+**^neurons in the short term. It can also inhibit the inflammatory response by inhibiting glycogen synthase kinase-3β (GSK-3β). It can also induce Akt and ERK to activate NF-κB and CREB transcription factors to regulate genes, promote the regeneration of BDNF neurons, and prolong the survival cycle (49). BDNF can bind to receptors on the facial nerve and promote axonal regeneration, and after electroacupuncture stimulation of rabbits with facial nerve injury, the expression of BDNF as well as the corresponding receptors was significantly up-regulated, which effectively repaired the damage to the facial nerve (50).

### 1.3 Acupuncture improves the immune response

Imbalances in the regulation of immune function play an important role in the formation, development, and outcome of peripheral facial palsy and are mainly associated with abnormal expression of immunoglobulins, immune cells, and immune molecules (51, 52). Facial paralysis is prone to occur after immunocompromise, or after excessive fatigue (53). Acupuncture can improve the body's immune function by regulating a variety of cells in the endogenous immune system (54). Studies have shown (55, 56) that the concentration of immunoglobulins in patients with facial paralysis is too high, and acupuncture treatment of facial paralysis can significantly down-regulate the levels of immunoglobulins IgA, IgG, and IgM. A study has also confirmed that warm acupuncture can up-regulate the expression of T lymphocyte subsets CD3+ and CD4+ in immune cells, down-regulate the expression of CD8+, and improve the clinical manifestations of facial paralysis (57). This is because T lymphocytes are also involved in the immune response process in peripheral facial paralysis (13, 58). The study also found that neutrophil-lymphocyte ratio (NLR), a key biomarker for evaluating immune function, was highly expressed in the serum of patients with peripheral facial paralysis (59). Acupuncture can down-regulate the serum neutrophil-to-lymphocyte ratio (NLR), and platelet-to-lymphocyte ratio (PLR) in patients with acute-phase facial neuritis (60).

### 1.4 Acupuncture inhibits oxidative stress

Oxidative stress is a state of imbalance between the oxidation system and the antioxidant defense system in the body, which is caused by excessive production of reactive oxygen species in the body, inducing apoptosis and damaging nerves (61). Overexpression of oxidative stress can damage nerves, so it may be a mechanism of facial nerve injury. The classical Mitogen-activated protein kinase/extracellular regulated protein kinase (MAPK/ERK) pathway is involved in the process of oxidative stress. In this pathway, extracellular stimuli activate and phosphorylate MEK, which in turn activates extracellular regulated protein kinases 1/2(ERK1/2), which is also phosphorylated to continue to activate downstream substrates and regulate cellular responses (62). It has been found that under hypoxia-induced oxidative stress environment, reactive oxygen species are increased in astrocytes, and activated transcription factor 1 (ATF-1) in the MAPK/ERK pathway may mediate ROS to inhibit thrombospondin-1(TSP-1) protein expression (63). ROS can bind to cell macromolecules and cause oxidative damage to the body through oxidation (64). Acupuncture can inhibit oxidative stress response (65). By stimulating specific acupoints, acupuncture can reduce the level of lipid peroxidation and activate the antioxidant enzyme system, to balance the oxidative stress state of the body (66). This process involves multiple pathways, including acupuncture regulating ROS production, affecting antioxidant enzyme pathways, repairing damaged biomolecules, and inhibiting apoptosis or autophagy (67). The rat model of acute lung injury confirmed that acupuncture inhibited the production of ROS by inhibiting the content of HO-1 and enhancing mitochondrial function, thereby protecting the damaged lung tissue (68). Acupuncture can also regulate gene expression, especially activate the antioxidant main regulatory factor Nrf2 (69), enhance SOD activity, inhibit oxidative stress, reduce oxygen free radical production, and reduce oxidative stress levels in inflammatory diseases (25). Acupuncture reduces the level of oxidative stress by increasing the expression of antioxidant enzymes. Acupuncture treatment significantly up-regulated the expression levels of antioxidant enzymes catalase (CAT), superoxide dismutase (SOD), and glutathione peroxidase (GSH-Px) in the depressive symptom model constructed by chronic unpredictable stress (CUMS) in rats (70). Acupuncture can also regulate the signal transmission process *in vivo* by blocking the MAPK/ERK signaling pathway, thereby effectively inhibiting oxidative stress (71). In addition, Acupuncture can modulate the expression of nitric oxide (NO), Malondialdehyde (MDA), and SOD in the body, which reflect the level of oxidative stress in patients with facial paralysis (72).

### 1.5 Acupuncture improves facial microcirculation

Facial microcirculation is also one of the current pathogenesiss of facial paralysis, and facial neuritis occurs when the facial nerve becomes ischaemic and oedematous (73, 74). Inflammatory tissues are generally hypoxic, and under hypoxic conditions, the regulation of NO on the stability of the hypoxia-inducible factor HIF-1α subtype is weakened, resulting in excessive activation and accumulation of HIF-1α, while continuous hypoxia will reduce the biological activity of NO (75, 76). NO belongs to vasodilator, and endothelin belongs to vasoconstrictor. Both of them regulate vasoconstriction and relaxation. Under pathological conditions, the ability of NO to inhibit the production and release of endothelin (ET) is weakened, and ET is released in large quantities, resulting in increased vascular resistance, slowed blood flow, and vascular microcirculation disorder (77). Acupuncture may significantly improve microcirculation by activating acupoints, regulating hemodynamics, affecting vasoactive substances, and adjusting hormone levels (78–80). According to relevant research, the key mechanism of acupuncture activating meridian-acupoints is to increase blood perfusion, thereby achieving the effect of improving microcirculation (81). In patients with peripheral facial paralysis in the acute stage, acupuncture at Hegu acupoints was examined by laser speckle technique, and the results showed that patients' facial perfusion was significantly elevated, confirming the effectiveness and importance of acupuncture at Hegu acupoints to improve facial microcirculation (82). A study has confirmed that acupuncture intervention in rats with ischemic facial paralysis can improve facial microcirculation by increasing the level of NO and reducing the level of ET in the body (83). It has also been shown (84) that the combination of acupuncture and medicine in the treatment of peripheral facial paralysis can promote the content of peripheral blood vascular endothelial progenitor cells (EPCs), which in turn promotes the regeneration and repair of facial blood vessels and improves facial microcirculation. In addition, it was found (85) that in patients with peripheral facial paralysis in the acute phase, the facial artery showed a significant decrease in blood flow velocity and elevated resistance compared to the healthy side, whereas a significant increase in blood flow velocity and a decrease in resistance of the facial artery appeared after needling the facial acupoints. So the blood flow velocity and flow resistance indices of the facial arteries can be used as potential biomarkers of peripheral facial palsy.

### 1.6 Acupuncture improves brain functional connectivity and structure

In the early stage of peripheral facial paralysis, brain function has abnormal connectivity (86). Acupuncture can activate the related brain regions that regulate peripheral facial never (87). Electroacupuncture activates functional areas such as the cerebellum, superior frontal gyrus, superior temporal gyrus, and precentral gyrus, resulting in enhanced signaling connectivity in these areas (88, 89). Not only that, early and precise acupuncture treatment for facial paralysis also promotes cortical reorganization in patients with facial paralysis (90). Currently, there is a relative lack of research utilizing computed tomography (CT) and functional magnetic resonance imaging (fMRI) to investigate changes in brain tissue and network connectivity in patients with peripheral facial paralysis treated with acupuncture and moxibustion. However, neuroimaging-based approaches hold significant potential for evaluating the effects of acupuncture on brain structure and functional connectivity in these patients. Further advancements in this field are expected to provide deeper scientific insights and identify novel biological markers for the acupuncture-based treatment of facial paralysis.

### 1.7 The biological specificity mechanism of acupuncture stimulating acupoints

#### 1.7.1 Specificity of acupoints

Acupoints are a combination of visceral and somatic afferents that activate sensory afferents over a small diameter area of the skin surface, releasing neuropeptides, which are thought to be the response points of neurogenic inflammation in the skin (91), Acupoints have close connection with the nervous system, muscles, and blood vessels, and when they receive different external stimulation such as acupuncture, electroacupuncture, and moxibustion, they produce the biomolecule adenosine (92), After adenosine binds to its receptor, it can effectively inhibit downstream pro-inflammatory cytokine secretion and exert anti-inflammatory effects by modulating the NF-kB signaling pathway (93). Not only that, external stimulation of acupoints actives somatic afferent nerve endings and regulates nerve conduction, but it also promotes the release of neuropeptides such as interleukin-1β (IL-1β), interleukin-6(IL-6), or immune cytokines from immune cells, which regulates immune function (94) and adjusts substances such as substance P (SP), and calcitonin gene-related proteins (CGRP) (95, 96). Therefore, the biospecific effects of acupoints and the optimization of acupuncture point combinations play a crucial role in the effectiveness of acupuncture in treating diseases like peripheral facial paralysis (97). Acupuncture treatment of peripheral facial paralysis often uses Dicang, Jiache, Yangbai, Xiaguan, Taiyang, Sibai, Chengjiang, Quanliao, Yingxiang, and Yifeng, these acupoints are commonly used in clinical consensus acupoints (98) (Table 2). These acupoints belong to different meridians and are primarily distributed on the face, with their main therapeutic effect being the regulation and unblocking of the meridians. The biological specificity of these points has been confirmed through various studies. For example, after electroacupuncture at Dicang in patients with facial paralysis, the signal connection of the brain region of the patient changed (99). Moreover, the corresponding anatomical position of Dicang is the orbicularis oris muscle, which can improve the symptoms of drooping mouth angle in patients with facial paralysis. The location of the Yifeng point is just at the outlet of the main trunk of the facial nerve, which directly stimulates the facial nerve (99, 100). Another study (101) confirmed that Taiyang point through Dicang, and Jiache point could play a role in improving facial paralysis by down-regulating by substance P (SP), vasoactive intestinal peptide (VIP), calcitonin gene-related peptide (CGRP). A study found that Yangbai (GB14), Sibai (ST2) and Jiache (ST6) have nerve fiber tissue in their local area, forming a regional nerve tissue network, and acupuncture and moxibustion as an external signal can stimulate these nerves, and then dominate the muscles and blood vessels around the acupoints (102). Further research showed that electroacupuncture at different facial acupoints induces varying levels of neural excitability, suggesting that the specificity of each acupoint may underlie its therapeutic mechanism (103). Therefore, this points to the importance of acupoint specificity and combination formulas in maximizing the therapeutic effects of acupuncture for conditions like peripheral facial paralysis.

**Table 2:** Common acupoints for peripheral facial paralysis.

**Commonly used acupoint for peripheral facial paralysis**	**Affiliated meridian**	**The function of acupuncture points**	**Reference**
Dicang (ST4)	Stomach meridian	Dredging meridians, dispelling wind and relieving pain	(124)
Jiache (ST6)	Stomach meridian	Dispersed wind heat, switch winding	(124)
Yangbai (GB 14)	Gallbladder meridian	Promote the operation of qi and blood	(125)
Xiaguan (ST 7)	Stomach meridian	Activating blood, detumescence and relieving pain	(126)
Taiyang	Extraordinary points	Clearing heat and detumescence, relieving pain and soothing collaterals	(124)
Sibai (ST2)	Stomach meridian	Loosen tendons ligament	(127)
Chengjiang (CV24)	Stomach meridian	Loosen tendons ligament	(128)
Quanliao (SI18)	Small intestine meridian	Detumescence Qufeng	(129)
Yingxiang (LI20)	Large intestine meridian	Wind clearing heat	(130)
Yifeng (TE17)	Sanjiao meridian	Wind dredging collaterals	(131)

#### 1.7.2 Selectivity of acupoints

The selectivity of acupoints for appropriate diseases also plays a very important role in the treatment of diseases. The acupoints used clinically to treat peripheral facial paralysis mostly belong to the stomach meridian of foot yangming and the large intestine meridian of hand yangming in the 12 meridians. This is because the stomach and large intestine meridians run through the face of the human body, and the acupoints have the effect of treating local diseases. From a traditional Chinese medicine (TCM) perspective, the Yangming meridian is abundant in qi and blood, which promotes the circulation and recovery of facial qi and blood. The qi here refers to an intangible, functional energy that flows through the meridians and can enter and exit the acupoints (104). The blood is tangible, traveling through the meridians and resuscitating the five organs, and the qi generates and pushes the blood flow (105). Additionally, some acupuncturists will cooperate with Hegu to treat peripheral facial paralysis. This is based on the traditional experience of traditional Chinese medicine. The Hegu acupoint is located on the hand Yangming large intestine meridian, at the midpoint on the radial side of the second metacarpal bone, between the first and second metacarpal bones on the dorsum of the hand (106). Hegu has a special therapeutic effect on facial and oral diseases. Modern research supports this practice, for the reason that needling bilateral Hegu acupoints improves facial blood perfusion and enhances facial microcirculation (107).

### 1.8 The biological effects of different acupuncture intervention times on the body

As an effective traditional Chinese medicine therapy, the timing of acupuncture intervention is very important in the treatment of peripheral facial paralysis. Acupuncture can be intervened within 24 h after the onset of peripheral facial paralysis, and this conclusion is supported by the clinical research results of Song and Mou (108). Because the onset time of peripheral facial paralysis and the starting time of remission directly affect its prognosis, the earlier the intervention, the better the speed and effect of recovery (109). That will reduce the possibility of recurrence of facial paralysis (110). However, there is some controversy about whether acupuncture intervention will aggravate facial nerve inflammation and edema in the early stage of the disease (1). In fact, acupuncture can relieve acute facial inflammation and edema by stimulating facial nerves and acupoints (111), promote facial blood circulation, improve neurotrophy, reduce nerve compression, and accelerate facial muscle function recovery (110). In addition, a systematic evaluation showed that the timing of acupuncture intervention for peripheral facial paralysis had a significant effect on recovery and that early acupuncture treatment was able to speed up recovery, improve prognosis, and reduce the occurrence of sequelae (112). A large randomized controlled clinical study showed that starting treatment within 3 days after the onset of facial paralysis can effectively promote nerve recovery and shorten the healing time, and waiting until the condition enters the recovery period has a more limited effect (113). In addition, several studies (114, 115) have recommended the use of acupuncture from the early stage of the disease to clarify the safety and efficacy of acupuncture in the acute phase of peripheral facial paralysis from the perspective of evidence-based medicine.

### 1.9 Other potential mechanisms

Studies have found that electroacupuncture can reduce the expression of cytokine signal transduction inhibitory protein-3(SOCS-3), thereby inhibiting the JAK-STAT pathway, which may also be an important mechanism of acupuncture intervention in peripheral facial paralysis (116, 117). In addition, the mechanical stimulation of acupuncture can also be considered as a mechanism for acupuncture treatment of peripheral facial paralysis. This is because the mechanical stimulation of acupuncture activates the mechanically sensitive neurons on the body surface, transduces the signal afferent nerve fibers to the central neurons, and then passes from the efferent nerve to the related pathways, and then regulates the body movement and sensation (118).

## 2 Conclusion and outlook

**Figure 2:**
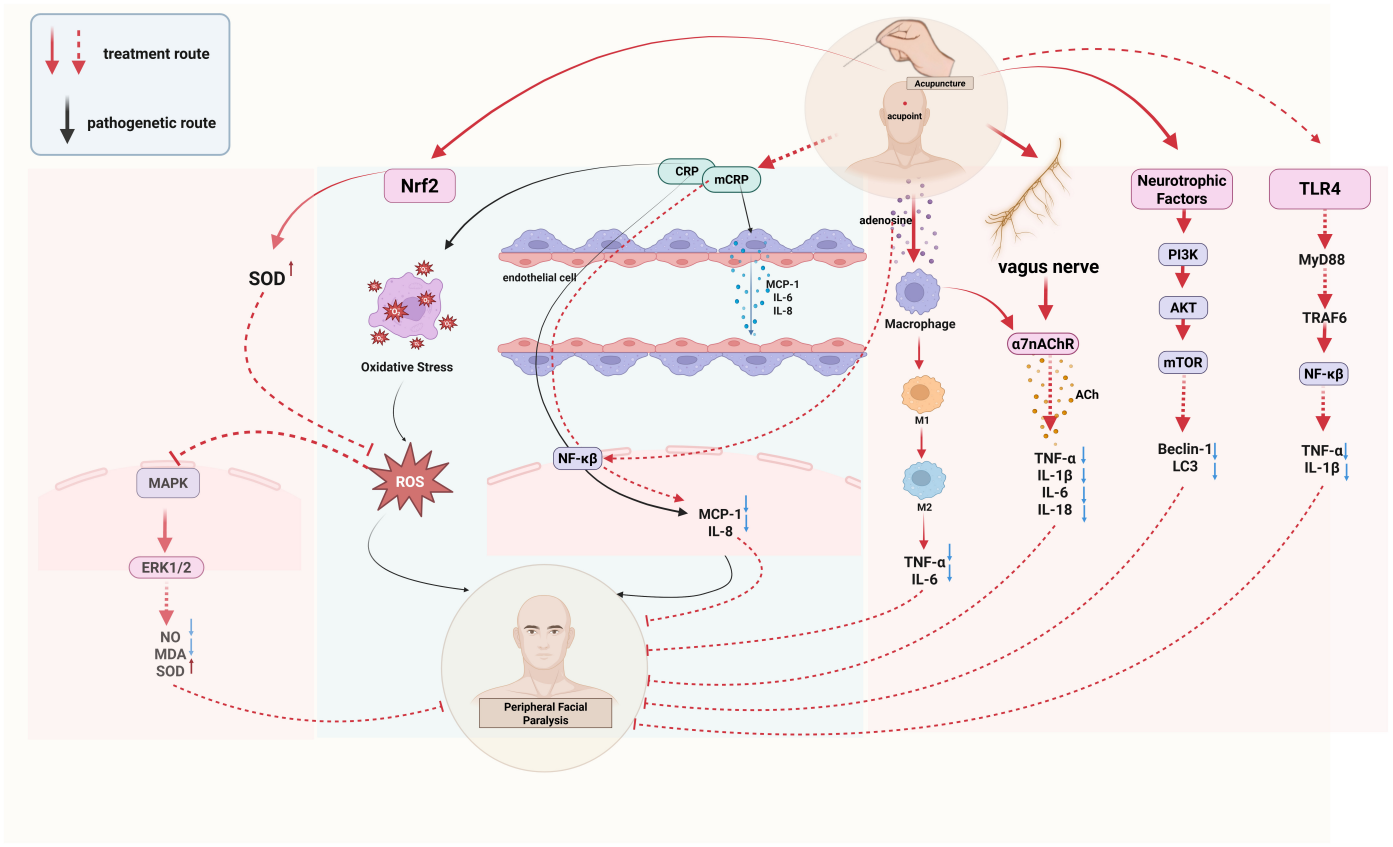
The biological process of acupuncture treatment of peripheral facial paralysis.

**Figure 3:**
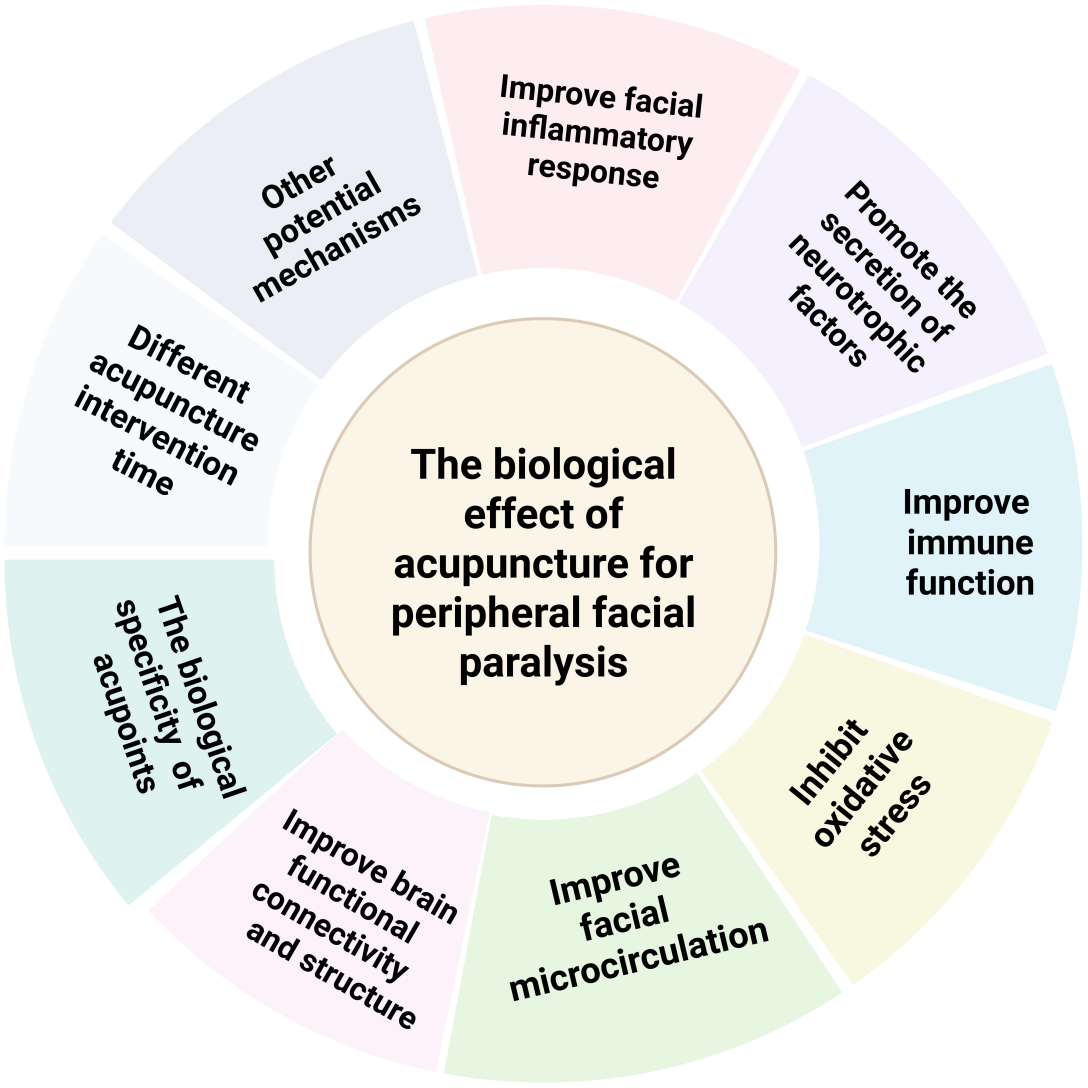
Biological effect of acupuncture intervention in peripheral facial paralysis.

This paper provides an overall overview of the changes in biological effects that occur in the body when acupuncture treats peripheral facial paralysis (Figure 2). The biological effects (Figure 3) of acupuncture intervention in peripheral facial paralysis are reflected in inflammatory mediators, immune system, nervous system, facial microcirculation, oxidative stress levels, abnormalities in structural and functional brain connectivity, and biological properties of acupoints. Current research primarily investigates acupuncture as an external stimulus that triggers one or more pathways, subsequently regulating downstream biological signaling to treat diseases. However, there is no clear answer to the current clinical research and experimental research on which pathway acupuncture has the most obvious regulatory effect. There are still some pathways such as JAK-STAT, Wnt/β-catenin signaling pathway (119), and PI3K/Akt signaling pathway (120), that have not been studied in depth, and repetitive studies are needed to get a clearer answer. In addition to the commonly discussed pathways associated with peripheral facial paralysis, a review (121) also highlights that the TGF-β/Smad signaling pathway can alleviate neuroinflammation by regulating cytokines. This suggests a potential direction for future research, where scholars may explore the use of acupuncture as a therapeutic approach to modulate peripheral facial paralysis. Furthermore, the Hippo signaling pathway has been found to interact with the production of reactive oxygen species and oxidative stress, thereby regulating cell differentiation, proliferation, survival and tissue regeneration (122). This finding provides a more comprehensive physiological and pathological mechanism direction for future research on acupuncture treatment of neuroinflammation, such as peripheral facial paralysis. In addition, recent studies have found that magnesium deficiency also seems to be a cause of peripheral facial paralysis (123), which may be a future research direction.

## 3 Discussion

At present, the research on the biological effect mechanism of acupuncture in the treatment of peripheral facial paralysis remains to be further studied. Future research should focus on revealing the biological basis of acupoint stimulation, exploring the biological changes brought about by different acupoint combinations, and examining the effect of treatment timing on biological effects. With the rise of individualized medical treatment, the personalization and precision of acupuncture treatment have become a new trend. In addition, interdisciplinary cooperation and technological innovation are crucial for the development of this field. It is necessary to strengthen cooperation with molecular biology, immunology, and other disciplines, and use advanced technologies such as metabolomics, proteomics, and magnetic resonance imaging to further explain the biological mechanism of acupuncture in the treatment of peripheral facial paralysis, and provide a scientific basis for acupuncture treatment of peripheral facial paralysis.

## References

[1] Wang Z Zhang J Zhang Z Liu Y Ren S Sun H. et al. Effects of acupuncture for Bell's palsy patients in the acute phase and its impact on facial nerve edema: a study protocol for a randomized, controlled trial. Front Neurol. (2024) 15:1327206. 10.3389/fneur.2024.1327206 38689877 PMC11058209

[2] Adour KK Byl FM Jr RLH Kahn ZM. Sheldon MI. The true nature of Bell's palsy: analysis of 1,000 consecutive patients. Laryngoscope. (1978) 88:787–801. 10.1002/lary.1978.88.5.787 642672

[3] Rajangam J Lakshmanan AP Rao KU Jayashree D Radhakrishnan R Roshitha B. et al. Bell Palsy: facts and current research perspectives. CNS Neurol Disord Drug Targets. (2024) 23:203–14. 10.2174/1871527322666230321120618 36959147

[4] Baugh RF Basura GJ Ishii LE Schwartz SR Drumheller CM Burkholder R. Clinical practice guideline: Bell's Palsy executive summary. Otolaryngol Head Neck Surg. (2013) 149:656–63. 10.1177/0194599813506835 24190889

[5] Dagenais F Neville C Desmet L Martineau S. Measuring the potential effects of mirror therapy added to the gold standard facial neuromuscular retraining in patients with chronic peripheral facial palsy: protocol for a randomized controlled trial. JMIR Res Protoc. (2023) 12:e47709. 10.2196/47709 37418307 PMC10362495

[6] Cuenca-Martínez F Zapardiel-Sánchez E Carrasco-González E La Touche R Suso-Martí L. Assessing anxiety, depression and quality of life in patients with peripheral facial palsy: a systematic review. PeerJ. (2020) 8:e10449. 10.7717/peerj.10449 33344085 PMC7718791

[7] Guo LH Ma Y Wu XD. AGREE-based evaluation and content analysis of evidence-based clinical practice guidelines for acupuncture-moxibustion. Zhongguo Zhen Jiu. (2019) 39:1223–8. 10.13703/j.0255-2930.2019.11.023 31724361

[8] Fujiwara T Hato N Kasahara T Kasuya D Shida K Tanabe M. Summary of Japanese clinical practice guidelines for Bell's palsy (idiopathic facial palsy) - 2023 update edited by the Japan Society of Facial Nerve Research. Auris Nasus Larynx. (2024) 51:840–5. 10.1016/j.anl.2024.07.003 39079445

[9] Yu G Luo S Zhu C Chen L Huang H Nie B. Global trends and performances of acupuncture therapy on Bell's Palsy from 2000 to 2023: a bibliometric analysis. J Pain Res. (2023) 16:2155–69. 10.2147/JPR.S401086 37397274 PMC10312334

[10] Hu L Sun J Pei L Chen L. Effect of acupuncture-moxibustion on idiopathic facial palsy at acute phase in the real world: a cohort study. Zhongguo Zhen Jiu. (2025) 45:133–8. 10.13703/j.0255-2930.20240826-k0001 39943750

[11] Cheng L Li XL Ying Y Du SH Zhang XD Guo W. Should acupuncture therapy be used for acute facial paralysis? A protocol for systematic review. Syst Rev. (2023) 12:43. 10.1186/s13643-023-02194-5 36918972 PMC10015935

[12] Shanmei Fang GF. Choice of the right moment in acupuncture treatment for peripheral facial paralysis. Chin Acupunct Moxibust. (2001) 7:21–22.

[13] Zhang W Xu L Luo T Wu F Zhao B Li X. The etiology of Bell's palsy: a review. J Neurol. (2020) 267:1896–905. 10.1007/s00415-019-09282-4 30923934 PMC7320932

[14] Yao Z Zhang Y Wu H. Regulation of C-reactive protein conformation in inflammation. Inflamm Res. (2019) 68:815–23. 10.1007/s00011-019-01269-1 31312858

[15] Khreiss T József L Potempa LA Filep JG. Loss of pentameric symmetry in C-reactive protein induces interleukin-8 secretion through peroxynitrite signaling in human neutrophils. Circ Res. (2005) 97:690–7. 10.1161/01.RES.0000183881.11739.CB 16123332

[16] Cayir S Hizli O Kayabasi S. Is C-reactive protein to albumin ratio an indicator of poor prognosis in Bell's palsy? Eur Arch Otorhinolaryngol. (2020) 277:115–9. 10.1007/s00405-019-05691-3 31620889

[17] Han C Xiao H Zhu Z Guan Y Su Y Ge M. Pressing moxibustion at Baihui(DU20)combined with acupuncture in the treatment of peripheral facial paralysis bright. Chin Med. (2024) 39:2034–6.

[18] Luo M Zhao F Cheng H Su M Wang Y. Macrophage polarization: an important role in inflammatory diseases. Front Immunol. (2024) 15:1352946. 10.3389/fimmu.2024.1352946 38660308 PMC11039887

[19] Song S An J Li Y Liu S. Electroacupuncture at ST-36 ameliorates DSS-induced acute colitis via regulating macrophage polarization induced by suppressing NLRP3/IL-1β and promoting Nrf2/HO-1. Mol Immunol. (2019) 106:143–52. 10.1016/j.molimm.2018.12.023 30610999

[20] Xu Y Cai L Chen Q Gan H. Effect of mouse nerve growth factor combined with acupuncture and moxibustion on pediatric facial neuritis and on the expression of reg/Th17 related cytokines. J Modern Integrat Med. (2017) 26:3892–5.

[21] Oh JE Kim SN. Anti-inflammatory effects of acupuncture at ST36 point: a literature review in animal studies. Front Immunol. (2021) 12:813748. 10.3389/fimmu.2021.813748 35095910 PMC8790576

[22] Halder N Lal G. Cholinergic system and its therapeutic importance in inflammation and autoimmunity. Front Immunol. (2021) 12:660342. 10.3389/fimmu.2021.660342 33936095 PMC8082108

[23] Nishio T Taura K Iwaisako K Koyama Y Tanabe K Yamamoto G. Hepatic vagus nerve regulates Kupffer cell activation via α7 nicotinic acetylcholine receptor in nonalcoholic steatohepatitis. J Gastroenterol. (2017) 52:965–76. 10.1007/s00535-016-1304-z 28044208

[24] Zhang L Wu Z Zhou J Lu S Wang C Xia Y. Electroacupuncture ameliorates acute pancreatitis: a role for the vagus nerve-mediated cholinergic anti-inflammatory pathway. Front Mol Biosci. (2021) 8:647647. 10.3389/fmolb.2021.647647 34055878 PMC8155617

[25] Li N Guo Y Gong Y Zhang Y Fan W Yao K. The anti-inflammatory actions and mechanisms of acupuncture from acupoint to target organs via neuro-immune regulation. J Inflamm Res. (2021) 14:7191–224. 10.2147/JIR.S341581 34992414 PMC8710088

[26] Gutierrez H Davies AM. Regulation of neural process growth, elaboration and structural plasticity by NF-κB. Trends Neurosci. (2011) 34:316–25. 10.1016/j.tins.2011.03.001 21459462 PMC3115056

[27] He A Ji R Shao J He C Jin M Xu Y. TLR4-MyD88-TRAF6-TAK1 complex-mediated NF-κB activation contribute to the anti-inflammatory effect of V8 in LPS-induced human cervical cancer SiHa. Cells Inflammation. (2016) 39:172–81. 10.1007/s10753-015-0236-8 26276130

[28] Xue X Liu Y Wang S-H Yuan H-Y Li J Pan S-A. Effect of electroacupuncture intervention on relieving pain and inflammation by suppressing TLR4/NF-κB signaling in rats with primary dysmenorrhea. Zhen Ci Yan Jiu. (2023) 48:63–70. 10.13702/j.1000-0607.20220224 36734500

[29] Lucas K Maes M. Role of the toll like receptor (TLR) radical cycle in chronic inflammation: possible treatments targeting the TLR4 pathway. Mol Neurobiol. (2013) 48:190–204. 10.1007/s12035-013-8425-7 23436141 PMC7091222

[30] Jian-Hui Y Li-Juan H Jian-Ping L Hui O Gao-Mei L Wen-Ting W. Effect of toll-like receptor 4/nuclear factor-kappa B pathway and its related inflammatory markers on peripheral facial neuritis. Dept Chin J Clini Pharmacol. (2017) 33:1317–20.

[31] Jiang Z Zhang X Zhang W Li D Xia F. Effect of electroacupuncture on TLR4/NF-κB signaling pathway in patients with peripheral facial neuritis. J North Sichuan Med College. (2020) 35:759–62.

[32] Escobar A Reis RL Oliveira JM. Nanoparticles for neurotrophic factor delivery in nerve guidance conduits for peripheral nerve repair. Nanomedicine (Lond). (2022) 17:477–94. 10.2217/nnm-2021-0413 35220756

[33] Staszkiewicz R Gralewski M Gładysz D Bryś K Garczarek M Gadzieliński M. Evaluation of the concentration of growth associated protein-43 and glial cell-derived neurotrophic factor in degenerated intervertebral discs of the lumbosacral region of the spine. Mol Pain. (2023) 19:17448069231158287. 10.1177/17448069231158287 36733259 PMC10071099

[34] Atzema C Goldman RD. Should we use steroids to treat children with Bell's palsy? Can Fam Physician. (2006) 52:313−4. PMC1479708 16572574

[35] Ohira Y. Effects of denervation and deafferentation on mass and enzyme activity in rat skeletal muscles. Jpn J Physiol. (1989) 39:21–31. 10.2170/jjphysiol.39.21 2724667

[36] Larkin LM Kuzon Jr WM Halter JB. Synergist muscle ablation and recovery from nerve-repair grafting: contractile and metabolic function. J Appl Physiol. (2000) 89:1469-76. 10.1152/jappl.2000.89.4.1469 11007584 PMC2714883

[37] Ozaki Y Ohashi K Otaka N Ogawa H Kawanishi H Takikawa T. Neuron-derived neurotrophic factor protects against dexamethasone-induced skeletal muscle atrophy. Biochem Biophys Res Commun. (2022) 593:5–12. 10.1016/j.bbrc.2022.01.028 35051783

[38] Pu H. Observation on the effectiveness of nerve growth factor in patients with acute facial paralysis. Chin J Pract Neurol. (2014) 17:32–4.

[39] Severini C. Neurotrophic factors in health and disease. Cells. (2022) 12:1. 10.3390/cells12010047 36611840 PMC9818562

[40] Ding Z Wu X. The mechanism of acupuncture in treatment of peripheral facial paralysis and research progress. Contemp Med. (2019) 25:176–9.

[41] Li XG Du JH Lu Y Lin XJ. Neuroprotective effects of rapamycin on spinal cord injury in rats by increasing autophagy and Akt signaling. Neural Regen Res. (2019) 14:721–7. 10.4103/1673-5374.247476 30632514 PMC6352584

[42] Yao J-P Feng X-M Wang L Li Y-Q Zhu Z-Y Yan X-Y. Electroacupuncture promotes functional recovery after facial nerve injury in rats by regulating autophagy via GDNF and PI3K/mTOR signaling pathway. Chin J Integr Med. (2024) 30:251–9. 10.1007/s11655-023-3610-7 38212498

[43] Fei J Gao L Li H-H Yuan Q-L Li L-J. Electroacupuncture promotes peripheral nerve regeneration after facial nerve crush injury and upregulates the expression of glial cell-derived neurotrophic factor. Neural Regen Res. (2019) 14:673–82. 10.4103/1673-5374.247471 30632508 PMC6352598

[44] Zang N Gu W Ma Y Wen H. Efficacy of staged acupuncture combined with warm acupuncture in the treatment of wind-cold type idiopathic facial nerve palsy in the acute stage and its effect on serum GDNF and NGF levels. Liaoning J Chin Med. (2023) 50:195–7.

[45] Tang C He J Jiang Y Tang S Zhou D. Clinical efficacy of alternating warm needle acupuncture combined with nerve growth factor from rats in the treatment of patients with acute idiopathic facial paralysis and its impact on facial nerve functionmedicine world. J Integrat. (2024) 19:111–5.

[46] Sleeman MW Anderson KD Lambert PD Yancopoulos GD Wiegand SJ. The ciliary neurotrophic factor and its receptor, CNTFR alpha. Pharm Acta Helv. (2000) 74:265–72. 10.1016/S0031-6865(99)00050-3 10812968

[47] Tong Z Wang X. A comparative study of the infuence of CNTF and NGF on facial nerve regeneration. J Modern Stomatol. (2002) 1:14–6.

[48] Sun Y Li Y Zhang W Peng X. Expressive level of CNTFR in electro-acupuncture for treating the acute facial nerve injury model. Liaoning J Trad Chin Med. (2011) 38:2271–2.

[49] Lima Giacobbo B Doorduin J Klein HC Dierckx RAJO Bromberg E de Vries EFJ. Brain-derived neurotrophic factor in brain disorders: focus on neuroinflammation. Mol Neurobiol. (2019) 56:3295–312. 10.1007/s12035-018-1283-6 30117106 PMC6476855

[50] Sun Z Wei Y Jiang G Kou J. Effects of electroacupuncture on the expression of BNDF mRNA in the nucleus of facial nerve in rabbits with peripheral facial nerve injury. Acupunct Stud. (2006) 4:204–7+57.

[51] Wang S Zhang J Wang H Dai L Xu B. Effects of acupuncture combined with acupoint catgut embedding on facial nerve and immune indexes in patients with peripheral facial paralysis. J Guangzhou Univer Chin Med. (2020) 37:1517–22.

[52] Li X-J Zhao Z-T Zhu T-T Zhao Y-K Yan X-K. Progress of the mechanism of acupuncture to promote repair of facial nerve injury. Acupunct Res. (2018) 43:60–2. 10.13702/j.1000-0607.170116 29383897

[53] Li D Ren Y Liang H Liang F. Professor Liang Fanrong′s experience in treating peripheral facial paralysis by phases based on the muscle meridian theory. World Chinese Med. (2022) 17:26324+9.

[54] Liu F Wang Y Lyu K Du X Zhou M Shi J. Acupuncture and its ability to restore and maintain immune homeostasis. QJM. (2024) 117:167–76. 10.1093/qjmed/hcad134 37318994

[55] Shuxin Z Qiang Y. Effect of acupuncture on facial nerve function and immune function in stroke patients with facial paralysis. Chinese J Trad Chin Med. (2018) 36:1972–4.

[56] Liang W. Clinical study on warming-needle moxibustion for peripheral facial paralysis. New Chin Med. (2023) 55:179–83.19563191

[57] Sun H Zhang B Qian H-H Chen Z-C. Effect of warm-needle moxibustion intervention on immune function and intestinal flora in patientsEffect of warm-needle moxibustion intervention on immune function and intestinal flora in patients. Acupunct Res. (2021) 46:592–7. 10.13702/j.1000-0607.200647 34369680

[58] Yang T Huang X Li F Liu T Wang Y Geng D. Correlation analysis of CD4+ effector memory T cells and NLR with idiopathic facial paralysis. J Med Res. (2021) 50:134–7+123.

[59] Yamamoto K Kurioka T Ohki M Ohashi K Harada Y Asako Y. Immune-nutritional status as a novel prognostic predictor of Bell's Palsy. Audiol Neurootol. (2022) 27:418–26. 10.1159/000524355 35512660

[60] Cui Q. Observations on the efficacy of acupuncture plus moxibustion for acute peripheral facial paralysis. Shanghai J Acupunct Moxibust. (2024) 43:59–65.

[61] Zhang B Shi H Cao S Xie L Ren P Wang J. Revealing the magic of acupuncture based on biological mechanisms: a literature review. Biosci Trends. (2022) 16:73–90. 10.5582/bst.2022.01039 35153276

[62] Lin Y Liu Q Chen C Chen W Xiao H Yang Q. Effect of acupuncture combined with hypothermia on MAPK/ERK pathway and apoptosis related factors in rats with cerebral ischemia reperfusion injury. Zhong Nan Da Xue Xue Bao Yi Xue Ban. (2017) 42:380–8. 10.11817/j.issn.1672-7347.2017.04.003 28490694

[63] Chen J-K Zhan Y-J Yang C-S Tzeng S-F. Oxidative stress-induced attenuation of thrombospondin-1 expression in primary rat astrocytes. J Cell Biochem. (2011) 112:59–70. 10.1002/jcb.22732 20524210

[64] Loh KP Huang SH De Silva R Tan BK Zhu YZ. Oxidative stress: apoptosis in neuronal injury. Curr Alzheimer Res. (2006) 3:327–37. 10.2174/156720506778249515 17017863

[65] Gao H Ding W. Effect and mechanism of acupuncture on endogenous and exogenous stem cells in disease treatment: a therapeutic review. Life Sci. (2023) 331:122031. 10.1016/j.lfs.2023.122031 37598978

[66] Zhao Y Zhou B Zhang G Xu S Yang J Deng S. The effect of acupuncture on oxidative stress: A systematic review and meta-analysis of animal models. PLoS ONE. (2022) 17:e0271098. 10.1371/journal.pone.0271098 36084019 PMC9462787

[67] Guo BJ Sun JH Pei LX. Research progress on mechanisms of acupuncture and moxibustion underlying improvement of oxidative stress. Zhen Ci Yan Jiu. (2024) 49:307–14. 10.13702/j.1000-0607.20221428 38500329

[68] Shi J Piao M Liu C Yang J Guan X Liu H. Electroacupuncture pretreatment maintains mitochondrial quality control via HO-1/MIC60 signaling pathway to alleviate endotoxin-induced acute lung injury. Biochim Biophys Acta Mol Basis Dis. (2024) 1870:167480. 10.1016/j.bbadis.2024.167480 39209235

[69] Hybertson BM Gao B Bose SK McCord JM. Oxidative stress in health and disease: the therapeutic potential of Nrf2 activation. Mol Aspects Med. (2011) 32:234–46. 10.1016/j.mam.2011.10.006 22020111

[70] Li J Wu X Yan S Shen J Tong T Aslam MS. Understanding the antidepressant mechanisms of acupuncture: targeting hippocampal neuroinflammation, oxidative stress, neuroplasticity, and apoptosis in CUMS rats. Mol Neurobiol. (2024). 10.1007/s12035-024-04550-5 39422855 PMC11880061

[71] Zhao J Li H Shi C Yang T Xu B. Electroacupuncture inhibits the activity of astrocytes in spinal cord in rats with visceral hypersensitivity by inhibiting P2Y(1) receptor-mediated MAPK/ERK signaling pathway. Evid Based Complement Alternat Med. (2020) 2020:4956179. 10.1155/2020/4956179 32184891 PMC7061128

[72] Pu Z Tang X Guo Y. Effect of acupuncture and moxibustion combined with moxa-wool moxibutsion on facial paralysis in children. Med Inform. (2022) 35:136–8.

[73] Cui H Chen Y Zhong W Yu H Li Z He Y. The asymmetric facial skin perfusion distribution of Bell's palsy discovered by laser speckle imaging technology. Clin Hemorheol Microcirc. (2016) 62:89–97. 10.3233/CH-152006 26444618

[74] Wu X Xie Z Lu M Dong G Ran H. Value of color Doppler ultrasound in evaluating efficacy for acupuncture treatment of peripheral facial paralysis. J Clini Ultrasound Med. (2018) 20:106–9.

[75] Cummins EP Keogh CE Crean D Taylor CT. The role of HIF in immunity and inflammation. Mol Aspects Med. (2016) 47–48:24–34. 10.1016/j.mam.2015.12.004 26768963

[76] Brüne B Zhou J. The role of nitric oxide (NO) in stability regulation of hypoxia inducible factor-1alpha (HIF-1alpha). Curr Med Chem. (2003) 10:845–55. 10.2174/0929867033457746 12678687

[77] Warner TD. Relationships between the endothelin and nitric oxide pathways. Clin Exp Pharmacol Physiol. (1999) 26:247–52. 10.1046/j.1440-1681.1999.03025.x 10081622

[78] Wang G Litscher D Tian Y Gaischek I Jia S Wang L. Acupoint activation: response in microcirculation and the role of mast cells. Medicines. (2014) 1:56–63. 10.3390/medicines1010056 28933377 PMC5532981

[79] Wang Q Zhang Q Lu F Hu H Zhu M. Trends in acupuncture therapy for microcirculation and hemorheology from 1998 to 2023: a bibliometric and visualized study. J Pain Res. (2024) 17:177–96. 10.2147/JPR.S441512 38223661 PMC10785693

[80] Kim S-Y Min S Lee H Cheon S Zhang X Park J-Y. Changes of local blood flow in response to acupuncture stimulation: a systematic review. Evid Based Complement Alternat Med. (2016) 2016:9874207. 10.1155/2016/9874207 27403201 PMC4923553

[81] Lin Y-Y Yao T-T Zheng L Hong X-B Xu D-H. Application of laser speckle contrast imaging technology to researches on acupuncture and microcirculation. Zhen Ci Yan Jiu. (2020) 45:513–6. 10.13702/j.1000-0607.190033 32643891

[82] Zhong W-z Cui H Chen Y Yang Z-x Yu H-b Rao X-d. Blood perfusion effect of acupuncture on acute facial paralysis observed by laser speckle technique. J Acupunct Tuina Sci. (2014) 12:160–4. 10.1007/s11726-014-0765-y

[83] Lv S Xuan L Yu B Wan Y Wei Y Li Y. Effect of thick-needle governor vessel superficial insertion on serum nitric oxide and endothelin contents in rats with ischemic facial paralysis. J Zhejiang Univer Tradit Chin Med. (2016) 40:274–7.

[84] Liu J Zhang L Liu F Duan T. Effects of “Qianzheng San” with warm acupuncture at Zusanli on endothelial progenitor cells from peripheral blood in patients with acute idiopathic facial palsy. Shanghai J Trad Chin Med. (2016) 50:50–2.

[85] Mao X. Ultrasonic observation on improving related indicators of facial artery after facial paralysis by facial acupuncture. Clini Res Trad Chin Med. (2019) 11:33–5.

[86] Li X Zhao C Liu Z Chen C Li Z Liang L. Review of fMRI research on mechanism of acupuncture treatment of facial paralysis. J Trad Chin Med. (2021) 36:2122–7.

[87] Seon C Lee DH Kwon BI Yu JS Park SK Woo Y. Neural mechanisms of acupuncture for peripheral facial nerve palsy: A protocol for systematic review and meta analysis. Medicine. (2023) 102:e33642. 10.1097/MD.0000000000033642 37145006 PMC10158860

[88] Tang H Wang H Xu H Han J. fMRI study on peripheral facial paralysis patients by electroacupuncture (EA) Hegu(LI4) on the left. Chin J Trad Chin Med. (2010) 28:964–6.

[89] Tang H-t Wang H Zhu B Xu H-b Han J-z. Electroacupuncturing acupoints of patients with peripheral facial paralysis:a functional MRI study. Chin Med Imag Technol. (2009) 25:1167–70.21739695

[90] Yu L-Q Ma H Cao L-Y Zhou Y-L. Noninvasive evaluation of acupuncture-induced cortical plasticity in advanced rehabilitation of facial paralysis. J Craniofac Surg. (2024) 35:2015–20. 10.1097/SCS.0000000000010544 39178417

[91] Fan Y Kim D-H Gwak YS Ahn D Ryu Y Chang S. The role of substance P in acupuncture signal transduction and effects. Brain Behav Immun. (2021) 91:683–94. 10.1016/j.bbi.2020.08.016 32956833 PMC7749828

[92] Li F He T Xu Q Lin L-T Li H Liu Y. What is the acupoint? A preliminary review of acupoints. Pain Med. (2015) 16:1905–15. 10.1111/pme.12761 25975413

[93] Varani K Ma H Cao LY Zhou YL. The role of adenosine receptors in rheumatoid arthritis. Autoimmun Rev. (2010) 10:61–4. 10.1016/j.autrev.2010.07.019 20691813

[94] Chen B Zhao X Li M-y Guo Y. Progress of researches and comments on promoters initiating effects of acupuncture stimulation of acupoints. Zhen Ci Yan Jiu. (2013) 38:511–4. 24579369

[95] Hsiao IH Liao HY Cheng CM Yen CM Lin YW. Paper-based detection device for microenvironment examination: measuring neurotransmitters and cytokines in the mice acupoint. Cells. (2022) 11:18. 10.3390/cells11182869 36139442 PMC9497130

[96] Yang Y Qu J-Y Guo H Zhou H-Y Ruan X Peng Y-C. Electroacupuncture at sensitized acupoints relieves somatic referred pain in colitis rats by inhibiting sympathetic-sensory coupling to interfere with 5-HT signaling pathway. Chin J Integr Med. (2024) 30:152–62. 10.1007/s11655-023-3565-8 38038835

[97] Xing JJ Zeng BY Li J Zhuang Y Liang FR. Acupuncture point specificity. Int Rev Neurobiol. (2013) 111:49–65. 10.1016/B978-0-12-411545-3.00003-1 24215917

[98] Zheng H Li Y Chen M. Evidence based acupuncture practice recommendations for peripheral facial paralysis. Am J Chin Med. (2009) 37:35–43. 10.1142/S0192415X09006631 19222110

[99] Zhang Z Li Z. Clinical study of electroacupuncture for peripheral facial paralysis in patients with left, right Dicang fMRI NSM-S15P type MRI. Clini J Acupunct. (2015) 31:32–4.

[100] Cheng KJ. Neuroanatomical basis of acupuncture treatment for some common illnesses. Acupunct Med. (2009) 27:61–4. 10.1136/aim.2009.000455 19502461

[101] Wang B Tan C Xu Q Huang J Wang D. Clinical study of puncturing Taiyang(EX-HN5) towards Dicang(ST4) and Jiache(ST6) clinical study of puncturing Taiyang(EX-HN5) towards Dicang(ST4) and Jiache(ST6). Shanghai J Acupunct. (2020) 39:1385–90.

[102] Wang J Cui J-J Xu D-S Su Y-X Liao J-Y Wu S. Sensory and autonomic innervation of the local tissues at traditional acupuncture point locations GB14, ST2 and ST6. Acupunct Med. (2022) 40:546–55. 10.1177/09645284221085579 35579008

[103] Yang L Zhang K Zhang W Zhuang Z. Correlation of the electric excitability treated with electroacupuncture at different acupoints and the prognosis of Bell's palsy. Chin Acupunct Moxibust. (2018) 38:1288–92. 10.13703/j.0255-2930.2018.12.010106 30672217

[104] Lee BH. A Perspective on the identity of the acupoint. J Acupunct Meridian Stud. (2024) 17:111–5. 10.51507/j.jams.2024.17.4.111 39205613

[105] Li WW Guo H Wang XM. Relationship between endogenous hydrogen sulfide and blood stasis syndrome based on the Qi-blood theory of Chinese medicine. Chin J Integr Med. (2013) 19:701–5. 10.1007/s11655-013-1567-7 23975135

[106] Peng Y-Y Peng Y Meng H-T Guo C. A brief discussion on theoretical basis and clinical application of “treatment of orofacial diseases by using Hegu(LI4)”. Zhen Ci Yan Jiu. (2021) 46:84–6. 33559432 10.13702/j.1000-0607.200413

[107] Wang S-Y Qu X-X Song X-J Li S-Y Ma H-M Zhang D. Blood perfusion in different facial acupoint areas and its changes after acupuncture stimulation of Hegu (LI 4) displayed by laser Doppler imager in healthy volunteers. Zhen Ci Yan Jiu. (2012) 37:482–7. 23383458

[108] Song GM Mou KJ. Effect of the acupuncture within 24 hour after the onset of peripheral facial paralysis. Asian J Surg. (2022) 45:3024. 10.1016/j.asjsur.2022.06.160 35850903

[109] Peitersen E. Bell's palsy: the spontaneous course of 2,500 peripheral facial nerve palsies of different etiologies. Acta Otolaryngol Suppl. (2002) 549:4–30. 10.1080/000164802320401694 12482166

[110] Choi Y Lee S Yang C Ahn E. The impact of early acupuncture on bell's palsy recurrence: real-world evidence from Korea. Healthcare. (2023) 11:3143. 10.3390/healthcare11243143 38132033 PMC10743002

[111] Wang Y Yu XY. Clinical observation of interventional opportunity for acupuncture treatment of acute facial neuritis. Zhongguo Zhen Jiu. (2019) 39:237–40. 10.13703/j.0255-2930.2019.03.003 30942007

[112] Jin D-D Ye J Guo M Zhou J-W. Efficacy of acupuncture-moxibustion on peripheral facial paralysis at different time points: a meta-analysis. Zhongguo Zhen Jiu. (2020) 40:664–8. 10.13703/j.0255-2930.20190721-k0003 32538021

[113] Li Y Li Y Liu L-a Zhao L Hu K-m Wu X. Acupuncture and moxibustion for peripheral facial palsy at different stages: multi-central large-sample randomized controlled trial. Zhongguo Zhen Jiu. (2011) 31:289–93. 21528591

[114] Chen J Shi H Gao W Li X Shu Y Wang Y. Effect of the staging comprehensive treatment with acupuncture-moxibustion on Bell's facial palsy in the acute stage. Zhongguo Zhen Jiu. (2024) 44:51–6. 10.13703/j.0255-2930.20230717-k0002 38191159

[115] Zhu P Sun M Tang X Zhang X Guo Y. Repetitive transcranial acupuncture stimulation combined with electroacupuncture in treatment of acute facial palsy with retroauricular pain: a randomized controlled trial. Zhongguo Zhen Jiu. (2024) 44:489–94. 10.13703/j.0255-2930.20231014-k0003 38764097

[116] Gao J Tang C-L Liu R-J Chen X-L Xie H Hou Y-X. Effect of different intensities of electroacupuncture stimulation on expression of SOCS-3 and PPAR-gamma mRNA in adipose tissues of obesity rats. Zhen Ci Yan Jiu. (2013) 38:31–4. 23650797

[117] Li X Yan X Cao Z Wei Y Zhao Z. Effect of different kinds of electroacupuncture on the expression of pJAK1 and pSTAT3 in rabbits with acute facial nerve injury. Zhen Ci Yan Jiu. (2018) 29:1264–6.

[118] Wang M Liu W Ge J Liu S. The immunomodulatory mechanisms for acupuncture practice. Front Immunol. (2023) 14:1147718. 10.3389/fimmu.2023.1147718 37090714 PMC10117649

[119] Xia W Zhu J Wang X Tang Y Zhou P Wei X. Overexpression of Foxc1 regenerates crushed rat facial nerves by promoting Schwann cells migration via the Wnt/β-catenin signaling pathway. J Cell Physiol. (2020) 235:9609–22. 10.1002/jcp.29772 32391604 PMC7586989

[120] Fei J Chen S Song X Liang Y Duan K Peng X. Exogenous GDNF promotes peripheral facial nerve regeneration in rats through the PI3K/AKT/mTOR signaling pathway. FASEB J. (2024) 38:e23340. 10.1096/fj.202301664R 38031959

[121] Kumari S Dhapola R Sharma P Nagar P Medhi B HariKrishnaReddy D. The impact of cytokines in neuroinflammation-mediated stroke. Cytokine Growth Factor Rev. (2024) 78:105–19. 10.1016/j.cytogfr.2024.06.002 39004599

[122] Amanda B Pragasta R Cakrasana H Mustika A Faizah Z Oceandy D. The hippo signaling pathway, reactive oxygen species production, and oxidative stress: a two-way traffic regulation. Cells. (2024) 13:1868. 10.3390/cells13221868 39594616 PMC11592687

[123] Demir CY Bozan N. Serum magnesium concentration in patients with bell's palsy: a case-control study. Ear Nose Throat J. (2024) 2024:1455613241266694. 10.1177/01455613241266694 39056518

[124] Wang B Tan C Xu Q Huang J Wang T. Clinical study on the treatment of peripheral facial paralysis by solar transdiaconal and buccal car and its effect on facial nerve conduction function. Shanghai J AcupunctMoxibust. (2020) 39:1385–90.

[125] Chen X Wang Y. Brief talk on Yangbai (GB 14). Zhongguo Zhen Jiu. (2012) 32:1084. 23301472

[126] Dong ZY Zhang BQ Guo XQ. Acupuncture combined with herbal-cake partitioned moxibustion is superior to routine acupuncture in the treatment of peripheral facial paralysis. Zhen Ci Yan Jiu. (2019) 44:131–5. 10.13702/j.1000-0607.180304 30945490

[127] Wang T Li Z Ge T Zhang M Yuan A Yang J. Summary of professor YANG Jun's experience for intractable facial paralysis. Zhongguo Zhen Jiu. (2017) 37:649–51. 10.13703/j.0255-2930.2017.06.021 29231509

[128] Qiu R-R Zhang H Zhao D-F Chen M-Y Li D Tan J. Acupoint selection rules of neurogenic dysphagia treated with acupuncture and moxibustion in ancient times. Zhongguo Zhen Jiu. (2020) 40:891–6. 10.13703/j.0255-2930.20190708-k0001 32869602

[129] Ma L Zeng J. Treatment of facial muscle spasm by acupuncture at opposite points in 40 cases. Chinese Acupunct Moxibust. (2021) 41:1398.

[130] Zhu XM Nie RF Ding RZ. Innovative application of Yingxiang (LI 20) and Neiyingxiang (EX-HN 9) in acupuncture verses. Zhongguo Zhen Jiu. (2014) 34:984–6. 25543431

[131] Xu L-W Song C-X Quan X-M Liu Y-L Wu S-B. Jingjin needling improves facial nerve function and psychosomatic function in Hunt facial para-lysis patients. Zhen Ci Yan Jiu. (2020) 45:330–3. 10.13702/j.1000-0607.190447 32333541

